# Comparative transcriptomic analysis revealed important processes underlying the static magnetic field effects on *Arabidopsis*

**DOI:** 10.3389/fpls.2024.1390031

**Published:** 2024-05-28

**Authors:** Xiujuan Zhou, Lin Zhang, Peng Zhang, Hang Xu, Jialei Song, Yafei Chang, Tiantian Cai, Can Xie

**Affiliations:** ^1^ High Magnetic Field Laboratory, Hefei Institutes of Physical Science, Chinese Academy of Sciences, Science Island, Hefei, China; ^2^ Science Island Branch of Graduate School, University of Science and Technology of China, Hefei, China; ^3^ Hainan Key Laboratory for Sustainable Utilization of Tropical Bioresource, College of Tropical Crops, Hainan University, Haikou, China; ^4^ Sanya Nanfan Research Institute of Hainan University, Hainan Yazhou Bay Seed Laboratory, Sanya, China; ^5^ Institute of Quantum Sensing, Zhejiang University, Hangzhou, China

**Keywords:** static magnetic field, plant growth, comparative transcriptomic analysis, iron homeostasis, oxidative stress

## Abstract

Static magnetic field (SMF) plays important roles in various biological processes of many organisms including plants, though the molecular mechanism remains largely unclear. Here in this study, we evaluated different magnetic setups to test their effects on growth and development on *Arabidopsis* (*Arabidopsis thaliana*), and discovered that plant growth was significantly enhanced by inhomogeneous SMF generated by a regular triangular prism magnet perpendicular to the direction of gravity. Comparative transcriptomic analysis revealed that auxin synthesis and signal transduction genes were upregulated by SMF exposure. SMF also facilitated plants to maintain the iron homeostasis. The expression of iron metabolism-related genes was downregulated by SMF, however, the iron content in plant tissues remains relatively unchanged. Furthermore, SMF exposure also helped the plants to reduce ROS level and synergistically maintain the oxidant balance by enhanced activity of antioxidant enzymes and accumulation of nicotinamide. Taken together, our data suggested that SMF is involved in regulating the growth and development of *Arabidopsis thaliana* through maintaining iron homeostasis and balancing oxidative stress, which could be beneficial for plant survival and growth. The work presented here would extend our understanding of the mechanism and the regulatory network of how magnetic field affects the plant growth, which would provide insights into the development of novel plant synthetic biology technologies to engineer stress-resistant and high-yielding crops.

## Introduction

The life on earth is evolved and developed in the context of the boundary conditions of Earth, including Earth’s gravity and magnetic field (geomagnetic field, GMF). Geomagnetic field is a natural component of the environment which has fundamental impacts on all living organisms, not only protecting the atmosphere from cosmic radiation and solar wind, but also acting on living systems and influencing many biological processes.

A variety of organisms can detect and utilize the geomagnetic field for different purpose. For example, magnetotactic bacteria can use GMF to align their bodies and navigate to their favored environments (Pazur et al., 2007; Moisescu et al., 2014; Lin et al., 2017). Various animals such as monarch butterflies, salmon and migratory birds can sense and use GMF information to navigate long distances, nesting or burrowing (Kirschvink et al., 2001; Wiltschko and Wiltschko, 2005, 2006; Guerra et al., 2014; Hore and Mouritsen, 2016; Qin et al., 2016; Mouritsen, 2018; Wan et al., 2021; Xie, 2022). However, unlike bacteria and animals can move over a long distance in response to GMF, plants generally don’t change their orientation once germinated. How GMF affects the development of plants remains a mystery and has attracted many researchers.

As GMF is a vector field that varies in space and time and can be characterized by different properties including inclination, polarity and intensity ranging from 25 to 65 microtesla (μT) (Finlay et al., 2010). To study the geomagnetic field effects on plants, SMF with different parameters has been applied to mimic or to strengthen GMF on different plant species. For example, SMF with different intensities was tested for the effects on plant growth, such as seed germination, root growth, flowering and so on. As reported previously, the flowering of *Arabidopsis* was delayed and the reproductive growth was inhibited under near-null magnetic field (NNMF) (Xu et al., 2021). Whereas at moderate SMF intensity (1 mT-1 T), the root growth of *Arabidopsis* was enhanced (Jin et al., 2019), the seed germination rate of tomato (*Solanum lycopersicum* L.) var. MST/32 (Abhary and Akhkha, 2023), maize (*Zea mays* L.) var. HQPM-1 (Torres et al., 2018) and mung bean (Mahajan and Pandey, 2014) were significantly increased. In contrast, at ultra-high SMF (e.g. 24.5 T SMF with - 150 T/m gradient), the germination rate of *Arabidopsis* seeds was significantly decreased (Xu et al., 2023). Besides the magnetic intensity, different type of magnets and magnetic setups, direction of the magnetic fields and the time of magnetic treatments, may have different effects on plants as well (Jin et al., 2019).

These findings clearly indicated that external magnetic fields played important roles in the growth and development of plants, however, the underlying mechanism is still not fully understood and currently on debate.

Many researchers believed that plants could perceive and respond to external magnetic fields using a mechanism similar to animal (e.g. Pigeon) magnetoreception, thus two key proteins, MagR and CRY, involved in animal magnetoreception were proposed and tested in plant magnetoreception (Ahmad et al., 2007; Chaves et al., 2011; Jin et al., 2019; Pooam et al., 2019; Parmagnani et al., 2022; Thoradit et al., 2023). Cryptochromes (CRYs) are a class of evolutionarily conserved flavin-containing blue light photoreceptors that are widely found in plants and animals. CRYs have been suggested to play essential roles in animal magnetoreception via radical-pair mechanism (Hore and Mouritsen, 2016). In plants, CRYs are mainly involved in the regulation of important growth and development processes such as photomorphogenesis, flowering time, and biorhythmia (Liu et al., 2016; Ponnu and Hoecker, 2022). Studies confirm that CRYs and auxin signaling pathway mediated increased root growth upon SMF treatment have also been found in *Arabidopsis* (Jin et al., 2019) and a weak 7 MHz radiofrequency magnetic field significantly reduces the bioreactivity of CRY1 to blue light in *Arabidopsis* seedlings (Müller and Ahmad, 2011; Albaqami et al., 2020). MagR is a highly conserved A-type iron and iron-sulfur cluster binding protein, and was identified as a novel magnetoreceptor in animals (Qin et al., 2016). It is certainly possible that the same protein(s) mediate magnetoreception in both plants and animals, but trigger different signaling pathways and lead to different phenotypes.

However, as an environmental factor, external magnetic fields can affect many biological processes in addition to magnetoreception. For example, metal homeostasis could also be affected by magnetic field, which may mediate the magnetic field effects (MFE) on plants as well. It has been reported that reduction of plant ion uptake and transport upon SMF treatment in *Arabidopsis* (Narayana et al., 2018). The accumulation of iron (Fe) and zinc (Zn) content, but not copper (Cu) and manganese (Mn), was also found to be affected by external magnetic fields (Islam et al., 2020).

Meanwhile, production of reactive oxygen species (ROS) upon magnetic field treatment could be another possible mechanism to explain the observed magnetic field effects on plants. Magnetic fields could disrupt the free radical process in cellular membrane, or affect the enzyme activities of catalase (CAT), superoxide dismutase (SOD), glutathione reductase (GR), glutathione transferase (GT), peroxidase (POD), ascorbate peroxidase (APX), and polyphenoloxidase (PPO), which all lead to change of ROS level. Experimental evidences have been accumulated in a number of different plant species, including sheep grass (*Leymus chinensis*), soybean, pea, maize, cucumber and broad bean (*Vicia faba* L.) (Xia and Guo, 2000; Baby et al., 2011; Polovinkina et al., 2011; Anand et al., 2012; Bhardwaj et al., 2012; Jouni et al., 2012). Different intensities of magnetic fields may have different effects on ROS homeostasis. SMF at low intensity has distinct impact in antioxidant-mediated reactions in plants to overcome possible redox imbalances (Maffei, 2014). Certain symplastic antioxidant enzyme activities and non-enzymatic antioxidant levels have been found increased in response to SMF at low intensities (Cakmak et al., 2012). It seems different SMF treatments may have different effects on ROS level in plants and play roles in the maintenance of ROS homeostasis.

Taken together, external magnetic fields could affect many aspects of plant growth and development, and the effects are dependent on the parameters of the magnetic fields and how we treat the plants with magnetic fields. Many biological processes could be involved in the perception and response to magnetic fields. In this study, we tested different magnetic setups for their effects on *Arabidopsis* seedlings and identified that triangular prism magnets have the most significant effects on growth. Comparative transcriptomic analysis and experimental validation were performed. Based on our study, iron homeostasis and oxidative stress and antioxidant balance seem to form the molecular basis of magnetic field effects in *Arabidopsis*. However, further investigation of how plants perceive magnetic fields is certainly required in the future.

## Materials and methods

### Plant growth, SMF treatment, and morphological analysis

**Figure 1:**
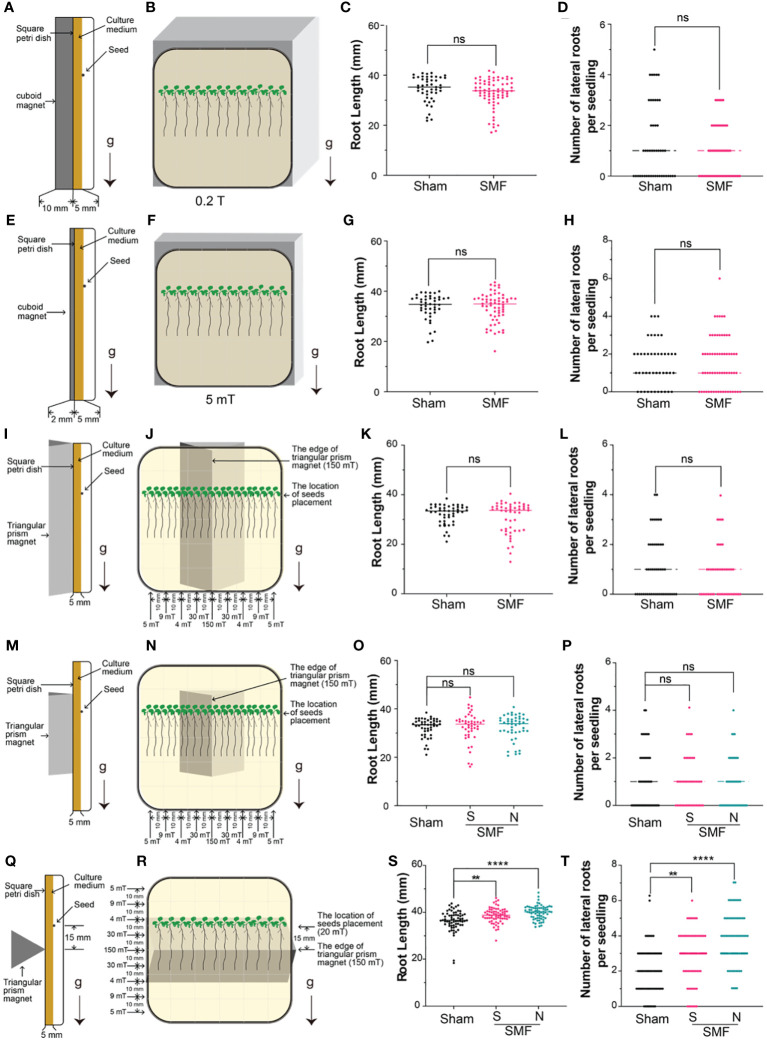
Screening of SMF conditions. The *Arabidopsis* seedlings were grown on a square petri dish with a magnet attached to the back of the dish, and the root length and lateral root number of 7-day-old seedlings were examined under different magnetic field conditions. **(A-H)** Weak gradient magnetic fields were generated by cuboid permanent magnets with different thickness, and the magnetic field strength can reach to approximately 200 mT **(A-D)** or 5 mT **(E-H)** on the surface of the south (S) pole. **(I-T)** Gradient magnetic fields were produced by regular triangular prism magnets with different length: 10 cm **(I-L)** or 3 cm **(M-P)** long with the edge parallel to the direction of gravity, or 10 cm long with the edge perpendicular to the direction of gravity **(Q-T)**. Arrows labeled “g” indicate the direction of gravity, while “S” and “N” respectively indicate that the edge of the magnet attaching to the square petri dish is the south pole (S) or the north pole (N). Data are shown means ± SD from three independent biological replicates (n = 20–24 seedlings), whereas the asterisk indicates significant (Student’s *t* test or one-way ANOVA, ns, no significance; **, *p*<0.01; ****, *p*<0.0001) differences between GMF and SMF.

The seeds of *Arabidopsis thaliana* plants Columbia ecotype (Col‐0) along with three iron homeostasis mutants, *irt1* (N676318), *irt2* (N666970) and *fro2* (N691003), were obtained from AraShare (https://www.arashare.cn/index). The *Arabidopsis* seeds were surface sterilized using sodium hypochlorite (8% v/v) for 8 minutes and then rinsed five times using sterilized distilled water. The surface-sterilized seeds were sown on half-strength Murashige and Skoog (MS) solid medium on square petri plates containing 0.8% agar. Plates were maintained for 3 days at 4°C for stratification, followed by germination in an Artificial Climatic Incubators (provided by XUNON) at 22°C under long-day conditions (16 h light, 8 h dark), with a light intensity of 80 to 90 μmol m^-2^ s^-1^. For iron treatment, 300 μM FeSO_4_ was added in half-strength MS solid medium. For SMF treatment, seedlings were maintained in half-strength MS medium with SMF were presented continuously for the entire period, from vernalization to germination and seedling growth. Five different magnetic treatment setups were provided by neodymium iron boron (NdFeB) N38 permanent magnets (Hefei Gaoshu Magnetic Materials, Hefei, China) with different size and shape, and schematic representations were shown in **Figure 1** and **Supplementary Figure S1**. For sham groups, seedlings were maintained in half-strength MS medium for the entire period in the absence of external magnetic field (only geomagnetic field presents), and non-magnetic aluminum 6061 (Al 6061) alloy blocks of the same size in the same experiment were used as a sham control of the magnets (Hefei Gaoshu Magnetic Materials, Hefei, China). After 7 days of seedling growth, the correlation between morphology was analyzed. The shoot area was measured using ImageJ software (Schneider et al., 2012) and the root length was measured with vernier calipers.

### Measurement of antioxidant enzyme activities and H_2_O_2_ concentrations

The activities of several antioxidant enzymes, including superoxide dismutase (SOD), catalase (CAT), ascorbate peroxidase (APX), peroxidase (POD) and the concentrations of H_2_O_2_, were quantified using assay kits purchased from Solarbio (Beijing, China), following the manufacturer’s instructions. The activities of SOD, CAT, APX, POD, and the concentration of H_2_O_2_ were calculated by measuring the absorbance at 560 nm, 240 nm, 290 nm, 420 nm and 415 nm, respectively using a microplate reader (Spectramax mini, Molecular Devices, USA). There are three separate biological duplicates in every experiment.

### Measurement of chlorophyll content

To extract the chlorophyll, 25 mg of 7-day-old fresh leaf tissue were soaked in 1 mL of 80% acetone in the dark until the residue turned white. The absorbance (A) of extracted chlorophyll was measured at 663 and 645 nm, respectively (Spectramax mini, Molecular Devices, USA). Total chlorophyll contents were calculated using the following equation: Chl a + Chl b=8.04 * A663 + 20.29 * A645, and the results were expressed as micrograms per gram fresh weight (Porra et al., 1989).

### Measurement of Fe, Cu and Zn content

Shoot and root samples were collected from *Arabidopsis* grown in half-strength MS in the presence or absence of 300 μM FeSO_4_, and exposed to SMF or none (Sham) for 7 continuous days. Collected samples were washed with 20 mM Tris and 5 mM EDTA for 10 minutes, and high‐performance liquid chromatography grade water for another 10 minutes, then dried in a ventilated oven (65°C) for 3 days, weighed, and used for ashing. The ash was digested 0.5 mL of 0.5 M nitric acid (Aladdin, USA) for 45 minutes at 75°C to a final volume of 0.4 mL. The solution was filtered through Millex^®^ GV 0.22 μm polyvinylidene fluoride membrane (PVDF) filter (Merck Millipore), and mineralized samples were transferred into polypropylene test tubes. Samples were diluted 20 times in MILLI-Q water, and the concentration of metal elements (Fe, Cu and Zn) was quantified by Thermo Scientific iCAP 7400 ICP-OES.

In addition, the iron (Fe) content was also determined using the ferrozine assay. Iron (II) reacts with ferrozine (0.1% (w/v) ferrozine in 50% (w/v) ammonium acetate) to form an intense purple complex that can be quantified spectrophotometrically at 562 nm using a microplate reader. Iron (Fe) content in plant tissue powder samples was quantified digestion treatment and two-fold dilution by reducing Fe with hydroxylamine hydrochloride (10% (w/v) HAHCl in 1 M HCl) and analyzed by ferrozine assay. Briefly, aliquots of the digest samples and HAHCl mixture (80 μL HAHCl and 20 μL samples) were incubated for 30 minutes at 37°C in the dark in a 96-well plate. Then, 100 μL of ferrozine was added to each well and incubated for another 15 minutes at 37°C in the dark. The iron-ferrozine complex was measured at 562 nm using a microplate reader (Spectramax mini, Molecular Devices, USA). Histogram and statistical analyzes were performed using GraphPad Prism software.

### Measurement of root surface activity of Cu (II) reductase and Fe (III) chelate reductase

To measure the root surface enzyme activities of Cu (II) and Fe (III) chelate reductases, intact roots of 7-day-old seedlings of *Arabidopsis* cultured on vertically placed square plates were used in this study. The measurement was performed as previously described (Yi and Guerinot, 1996; Bernal et al., 2012). Briefly, for the measurement of Fe (III) chelate reductase activity, the assay solution containing 0.1 mM Fe (III) NaEDTA and 0.3 mM Ferrozine [3-(2-pyridyl)-5,6-diphenyl-1,2,4-triazine-49,499-disulfonic acid] (Aladdin, USA); For the measurement of Cu (II) reductase activity, the assay solution containing of 0.2 mM CuSO_4_, 0.6 mM Na_3_citrate, and 0.4 mM BCDS (Sigma-Aldrich) were prepared in ultrapure water. Then, roots from 20 seedlings were pooled together and submerged in 1 mL of solution for each reductase assay in the dark and at room temperature. After 30 minutes for Fe (III) chelate reductase and 20 minutes for Cu (II) reductase, the UV-Vis absorbance of assay solutions were measured at 562 nm for the Fe (II) Ferrozine complex and at 483 nm for the Cu (I) BCDS complex, respectively. The activities were calculated followed the procedure published before (Welch et al., 1993), with the extinction coefficients of 28.6 mM^-1^ cm^-1^ for the Fe (II) Ferrozine complex and 12.25 mM^-1^ cm^-1^ for the Cu (I) BCDS complex.

### Metabolite profiling

Three biologically replicated samples from 7-day-old seedlings were used for metabolite analysis. Shoot and root samples (50 mg for each sample) were frozen and crushed with liquid nitrogen. Then, extracted using a 400 μL solution of methanol/water (4:1) and followed by ultrasound at 40 kHz for 30 minutes at 5°C. The mixture was placed at -20°C for 30 minutes to pellet the proteins and then centrifuged at 13,000 g at 4°C for 15 minutes. The obtained supernatants were filtered through Millex^®^ GV 0.22 μm PVDF filter (SCAA-104, 0.22 μm pore size; ANPEL, Shanghai, China) before LC-MS analysis. Analysis of the samples was completed as described previously using relative quantification method for widely targeted metabolites (Chen et al., 2013). A pooled quality control (QC) sample was prepared by mixing equal volumes of all samples and tested in the same manner as the analytical samples.

### Total RNA extraction and transcriptome sequencing

Total RNA was extracted using TRIzol^®^ Reagent (Plant RNA Purification for plant tissue) according to the manufacturer’s instructions (Invitrogen) and genomic DNA was removed using DNase I (TaKara). The integrity and purity of the total RNA was assessed by 2100 Bioanalyser (Agilent Technologies) and quantified using the ND-2000 (NanoDrop Technologies). Only high-quality RNA samples (OD260/280 = 1.8~2.2, OD260/230 ≥ 2.0, RIN ≥ 6.5, 28S:18S ≥ 1.0, >1 μg) were used to construct sequencing library.

RNA-seq transcriptome library of 7-day-old *Arabidopsis* shoots and roots were prepared following TruSeqTM RNA sample preparation Kit from Illumina (San Diego, CA) using 1 μg of total RNA. Briefly, messenger RNA was isolated according to polyA selection method by oligo(dT) beads and then fragmented by fragmentation buffer. Then double-stranded cDNA was synthesized using a SuperScript double-stranded cDNA synthesis kit (Invitrogen, CA) with random hexamer primers (Illumina). After that, the synthesized cDNA was subjected to end-repair, phosphorylation and “A” base addition, following Illumina’s library construction protocol. Libraries were size-selected on 2% low-range Ultra Agarose for cDNA fragments of 300 bp cDNA target fragments, and phusion DNA polymerase (NEB) was then used to amplify the target cDNA fragments for 15 cycles of PCR. After quantified by TBS380, the paired-end RNA-seq sequencing library was sequenced with the Illumina HiSeq xten/NovaSeq 6000 sequencer with a read length of 2 × 150bp.

### Transcriptomic analysis

The raw paired end reads were trimmed, and quality controlled by SeqPrep (https://github.com/jstjohn/SeqPrep) and Sickle (https://github.com/najoshi/sickle). Clean reads were then aligned to reference genome separately with orientation mode using HISAT2 (http://ccb.jhu.edu/software/hisat2/index.shtml) software (Kim et al., 2015). The mapped reads of each sample were assembled by StringTie (https://ccb.jhu.edu/software/stringtie/index.shtml?%20t=example) in a reference-based approach (Pertea et al., 2015).

To identify DEGs (differential expression genes) between two different samples (with different treatments), the expression level of each transcript was calculated based on the transcripts per million reads (TPM) method. RSEM (http://deweylab.biostat.wisc.edu/rsem/) (Li and Dewey, 2011) was used to quantify gene abundances. Differential expression analysis was performed using the DESeq2 (Love et al., 2014), DEGs with |log2FC| > 1 and Q value ≤ 0.05 were considered to be significantly DEGs. The GO analysis was conducted with DEGs by using online David website (https://david.ncifcrf.gov/tools.jsp). R software was used to draw the graph and TBTools was used draw the heatmap (Chen et al., 2023). Visualization of the network files to show the interaction of GO term and related genes was done using Cytoscape (v3.7.2) software (Shannon et al., 2003).

### Statistical analysis

In this study, data were presented as means ± standard errors. Number of replicates and statistical methods can be found in the corresponding figure legends. The Student’s *t* test was used to determine statistical significance between two groups. For multiple group comparisons, the data were analyzed by one-way analysis of variance (ANOVA). Asterisks indicate significant *, *p*<0.05; **, *p*<0.01; ***, *p*<0.001; ****, *p*<0.0001.

## Results

### SMF promotes the root growth of *Arabidopsis*

Change of root growth upon magnetic field treatment in a number of different plants has been reported previously, suggesting that root might be an ideal system to validate the magnetic field effects on plants (Maffei, 2014; Jin et al., 2019). Since different magnetic fields seem to impact plants differently as well, we explored the magnetic field effects on *Arabidopsis* using five different magnets, as shown in **Figure 1**. *Arabidopsis* seedlings were grown vertically in square petri dishes, with magnets closely attached to the back of dish for the entire period. Root length and number of lateral roots were measured and compared for each condition.

Weak gradient magnetic fields generated by cuboid permanent magnets with different heights were firstly tested for their MFE on *Arabidopsis* root growth, and non-magnetic aluminum alloy blocks of the same size in each experiment were used as a sham control of the magnets. Considering that the size of square petri dishes is 9 × 9 cm, and the magnets are 10 × 10 cm, therefore, the magnetic field intensity in the area while plant growing is almost uniform. Two conditions were tested: one cuboid neodymium iron boron (NdFeB) N38 permanent magnets (length × width × height: 10 × 10 × 0.2 cm) produce a magnetic field around 5 mT, and the other NdFeB N38 magnets (length × width × height: 10 × 10 × 1 cm) produce a magnetic field around 200 mT at the surfaces of the north (N) and south (S) poles. The magnets were attached to the back of petri dishes in our experiment to test the magnetic field effects on plants. It is important to point out that since seeds were planted and the roots were growing on the surface of solid medium in petri dish, which is about 5 mm away from the magnet surface (as illustrated in **Figures 1A, E, I, M, Q**), thus we also measured the magnetic intensity at 5 mm above the magnets, which may represent more precisely the actual magnetic field stimulation on the plants. Therefore, a NdFeB N38 permanent magnets (length × width × height: 10 × 10 × 0.2 cm) produce a magnetic field around 5 mT at the surfaces of magnet, and 4 mT on plants growing on solid medium in petri dish, which is 5 mm above the magnetic surface (**Supplementary Figure S1B**). And a thicker NdFeB N38 magnets (length × width × height: 10 × 10 × 1 cm), which produce around 200 mT magnetic field at the surfaces of magnet, and 150 mT on plants growing on solid medium in petri dish (**Supplementary Figure S1A**). No significant difference in root length and number of lateral roots were observed upon magnetic field treatment for these two conditions with different magnetic intensity (**Figures 1A-H**).

High gradient magnetic fields were then tested by using regular triangular prism magnets with different lengths (base × height × length: 2.5 × 2 × 3 cm, or 2.5 × 2 × 10 cm) and different placement pattern (parallel or perpendicular to gravity), as shown in **Figures 1I-T**. Firstly, we placed the regular triangular prism vertically with the edge parallel to the direction of gravity (**Figures 1I-P**). Both the 3 cm and 10 cm long regular triangular prism magnets produce around 200 mT magnetic field on the edge of the south (S) pole. The gradient magnetic fields generated by these triangular prism magnets have the highest magnetic field intensity measured at the edges of the prism, approximately 150 mT about 5 mm above the edge. As the distance away from the edge increases, the magnetic field intensity sharply decreases. At a distance of 1 cm from the edge and 5 mm above the magnets where plants were growing on culture medium, the magnetic field measures 30 mT, and at 2 cm it measures 4 mT, at 3 cm it measures 9 mT, and at 4 cm it measures 5 mT (which is close to the edge of the petri dish) (**Figures 1J, N, R**, **Supplementary Figure S1C, D**). No significant difference in root length and number of lateral roots were observed upon magnetic field treatment for this placement (the edge of triangular prism parallel to the direction of gravity) for both 3 cm and 10 cm length magnets (**Figures 1I-P**). Lastly, we placed the 10 cm long triangular prism magnet perpendicular to the direction of gravity to explore its effect on *Arabidopsis* seedlings (**Figures 1Q, R**) and found the root growth and lateral roots number were significantly enhanced upon magnetic field stimulation in both N pole and S pole treatments (**Figures 1S, T**).

Collectively, the data suggested the MFE not only relied on different magnets, but also depended on how the magnets were placed. Therefore, we applied magnetic field stimulations in later experiments with the magnetic field setup as shown in **Figures 1Q, R**.

### SMF rescued iron stress phenotypes in *Arabidopsis*

**Figure 2:**
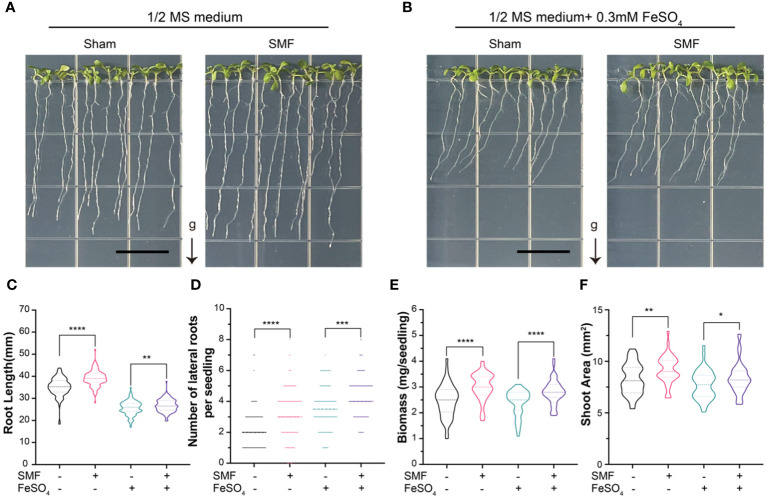
The growth of *Arabidopsis* seedlings upon SMF treatment, iron stress or the combination of SMF treatment and iron stress. **(A, B)** Representative images of the seedlings after 7 days of SMF treatment **(A)**, or the combination of SMF treatment and iron stress **(B)** and their controls. **(C-F)** The seedlings were subjected to analyses including total root length **(C)**, number of lateral roots **(D)**, fresh biomass of the whole plant **(E)** and relative shoot areas **(F)**. Data are means ± SD of three independent experiments (n = 24 seedlings), whereas the asterisk indicated significant difference tested by one-way ANOVA, *, *p*<0.05; **, *p*<0.01; ***, *p*<0.001; ****, *p*<0.0001.

Iron is an essential element for plant growth and development, and probably the most studied form of biomagnetism originates. Therefore, we further explored the MFE in the presence and absence of supplemented iron. Accordingly, all *Arabidopsis* plants grew under 4 different conditions: Sham, SMF, Sham with Fe supplemented, and SMF with Fe supplemented. Representative images of plants after treatments were taken (**Figures 2A, B**). Root length, number of lateral roots, biomass and relative shoot area were measured and compared (**Figures 2C-F**).

The results showed that the root lengths, biomass and relative shoot area were significantly decreased when iron was supplemented in the medium, indicating the growth of *Arabidopsis* was inhibited by severe iron stress on plants (**Figures 2C, E, F**). However, the number of lateral roots was higher than those conditions without iron stress (**Figure 2D**), suggesting lateral root growth could be one way for plants to respond to the iron stress.

Interestingly, the root lengths, amount of lateral root, biomass and relative shoot area are all significantly increased upon magnetic field treatment, compared with the sham groups in all conditions. It is important to point out that the phenotypes caused by iron stress in *Arabidopsis* seedlings appeared to be partially rescued by SMF treatment. For example, the iron stress induced decreased root lengths, biomass and relative shoot area, but all these phenotypes were increased significantly upon SMF treatment, though still lower than those in the absence of iron stress (**Figures 2C, E, F**). Meanwhile, chlorophyll contents showed no significant difference after SMF treatment, but increased after the addition of Fe, and then decreased after SMF treatment (**Supplementary Figure S2**).

In all, SMF stimulation promoted the growth of *Arabidopsis* seedlings, and iron stress inhibited plant growth. When we combine the SMF treatment and iron stress conditions together, SMF treatment could partially rescue the iron stress caused growth inhibition in *Arabidopsis* seedlings. To elucidate the possible underlying mechanism of MFE in *Arabidopsis* and the connection between MFE and iron metabolism, a comparative analysis of the gene expression profiles was performed.

### Comparative transcriptomic analysis

**Figure 3:**
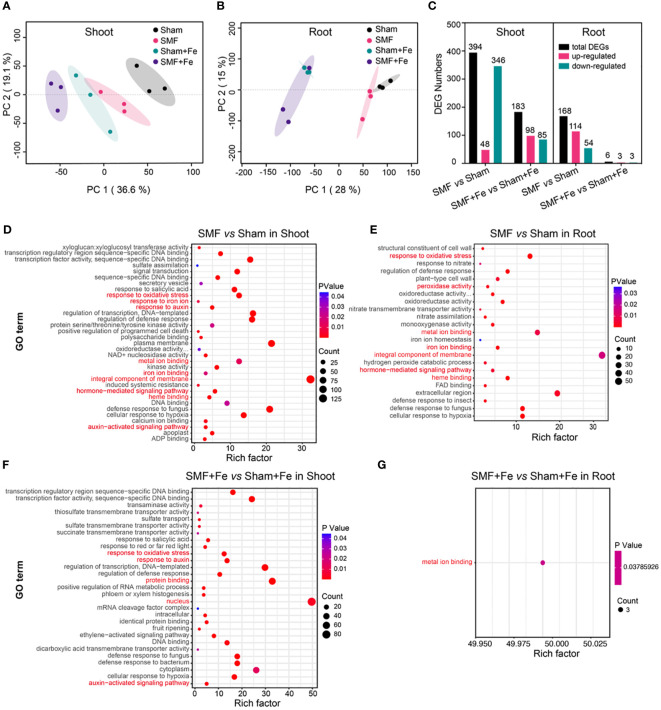
Comparative transcriptomic analysis of *Arabidopsis* upon SMF treatment, or iron stress, or the combination of SMF treatment and iron stress. **(A, B)** Principal component analysis (PCA) based on transcriptomic data of Shoot **(A)** and Root **(B)** in different conditions. The X-axis represents the first principal component (PC1), the Y-axis represents the second principal component (PC2). **(C)** Histogram showing the number of up- and downregulated differentially expressed genes (DEGs) identified in different conditions. **(D-G)** GO enrichment analysis of DEGs in the Shoot **(D, F)** and Root **(E, G)** between SMF and GMF, or between SMF with Fe and GMF with Fe, respectively. The X-axis is the rich factor, and the Y-axis represents the name of GO term. The bubble size represents the number of DEGs involved. The bubbles color indicates the enrichment degree of the pathway. The rich factor refers to the ratio of the number of genes enriched in the GO term to the number of annotated genes.

Considering magnetic field may have different effects on different parts of the same plant, *Arabidopsis* shoot and root samples were prepared from each condition for RNA sequencing separately. A total of 166.76 Gb clean data were generated from the 24 samples (**Supplementary Table S1**). The high-quality reads were assembled into 27,221 genes with 58,040 transcripts based on the TAIR10, including 46,345 known transcripts and 11,695 novel transcripts that were annotated in Nr, SwissProt, Pfam, COG, and KEGG databases (**Supplementary Table S2**). All raw sequences were cataloged in the NCBI Sequence Read Archive under accession number PRJNA1071296. Principal component analysis (PCA) was performed and showed that Sham, SMF, Sham with Fe supplemented, and SMF with Fe supplemented were the main drivers of gene expression in shoot and root, respectively (**Figures 3A, B**). Biological replicates were grouped together, and notably, one of the samples in SMF with Fe treatment was found to be similar to the samples in Sham with Fe, possibly due to the RNA quality issues.

In total, 394 differentially expressed genes (DEGs) in the shoots were identified in SMF treatment group compared with sham group. Among all these DEGs, 48 genes were upregulated and 346 genes were downregulated. 183 DEGs (98 upregulated and 85 downregulated) in the shoots were identified in SMF with Fe group compared with sham with Fe group. Whereas in roots, 168 DEGs (114 upregulated and 54 downregulated) between SMF and sham conditions and 6 genes (3 upregulated and 3 downregulated) between SMF with Fe and sham with Fe conditions were identified (**Figure 3C**).

To identify the processes enriched in significant DEGs, the identified DEGs were assigned to GO annotations, including biological processes (BP), molecular functions (MF), and cellular compartments (CC). As a result, 132 annotated GO items were significantly enriched (Padjust < 0.05) in the shoots between SMF and sham (**Figure 3D**), and 85 in the shoots between SMF with Fe and sham with Fe, respectively (**Figure 3E**). In the roots, 52 and 1 annotated GO terms were significantly enriched (Padjust < 0.05) between SMF and sham, and between SMF with Fe and sham with Fe, respectively (**Figures 3F, G**). Most DEGs were enriched in the biological processes such as “response to oxidative stress”.

In the shoots under SMF *vs.* sham conditions, “integral component of membrane” was significantly enriched. Interestingly, many DEGs were involved in the biological processes such as auxin metabolism, including auxin-activated signaling pathway (GO:0009734), response to auxin (GO:0009733), and hormone-mediated signaling pathway (GO:0009755). Additionally, antioxidant metabolism and metal ion metabolism were also enriched in our study, including heme binding (GO:0020037), response to oxidative stress (GO:0006979), metal ion binding (GO:0046872), iron ion binding (GO:0005506), and response to iron ion (GO:0010039) (**Figure 3D**). In the roots, similarity GO terms were enriched, with a specific enrichment in iron ion homeostasis (GO:0055072) (**Figure 3E**). However, in the comparison of SMF with Fe *vs.* sham with Fe conditions, “nucleus” and “protein binding” were significantly enriched in shoot (**Figure 3F**). Similarly, biological processes such as in response to auxin, the auxin-activated signaling pathway, and the response to oxidative stress were enriched (**Figure 3F**). In the roots, only metal ion binding was enriched in the comparison of SMF with Fe *vs.* sham with Fe conditions (**Figure 3G**). These results suggested that regulation of auxin pathway, iron homeostasis, and oxidative stress pathway could be involved in how plants respond to the SMF stimulation.

### Auxin biosynthesis and signal transduction involved in *Arabidopsis* responding to SMF

Phytohormone dynamics play essential roles in the perception and response to environment stress in plants. Auxin/Indoleacetic acid (Aux/IAA) biosynthesis and signal transduction participate in plant growth and development and were identified to respond to the SMF stimulation and iron stress in our study.

**Figure 4:**
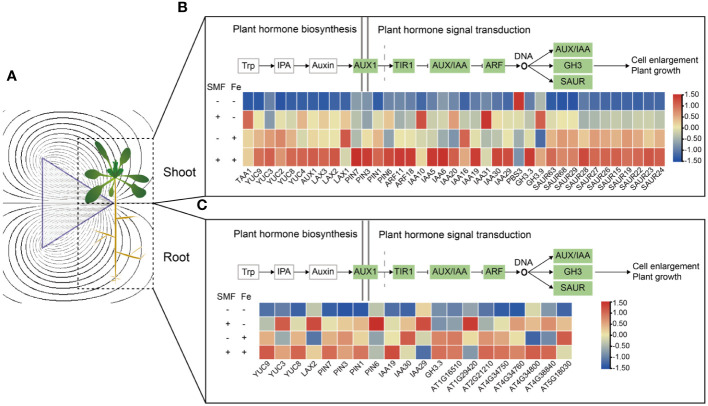
Transcriptional changes of DEGs involved in the plant hormone auxin biosynthesis and the auxin signal transduction in shoots and roots. **(A)** Schematic diagram of a magnetic field acting on *Arabidopsis* seedling. **(B, C)** Auxin biosynthesis and signal transduction gene expression in shoots **(B)** and roots **(C)**.

It is known that several gene families are involved in IAA biosynthesis, including YUCCA family and tryptophan aminotransferases, which catalyze the conversion of the Trp-derivative, TAM, to N-hydroxyl-tryptamine, and the transamination of Trp to form IPA, respectively. The *Arabidopsis* genome encodes four high-affinity auxin influx carriers (AUX1/LAX) that are involved in auxin uptake, while the PIN proteins are critical components of auxin efflux (Vanneste and Friml, 2009). Additionally, Gretchen Hagen 3 (GH3), small auxin-up RNA (SAUR), and Aux/indole-3-acetic acid (Aux/IAA) are involved in IAA signal transduction. Here in this study, we found that most IAA biosynthesis and signal transduction genes except *PBS3* were up-regulated after SMF exposure, such as *TAA1*, *YUC4*, *GH3.9*, which have high expression in the shoot (**Figures 4A, B**). In addition, when Fe was added, the expression of IAA biosynthesis and signal transduction genes was further upregulated in the shoot, no matter in the presence or absence of SMF stress (**Figure 4B**). This is consistent with previous studies showing that when plants were subjected to heavy metal stress, the stress was mitigated by adjusting auxin synthesis and transport (Moeen-Ud-Din et al., 2023). Exogenous IAA can reduce the shortened root phenotype caused by Zn by affecting auxin accumulation, and *Arabidopsis* mutants with defective auxin synthesis or transport are more susceptible to Zn (Wang and Yang, 2021). Overall, the combination of SMF treatment and iron stress resulted in a highest expression level. The same pattern of expression profile was observed in the roots, where *YUC3*, *LAX2*, *PIN6*, and *IAA29* were upregulated by SMF exposure (**Figures 4A, C**). Thus, the SMF promoted *Arabidopsis* seedling growth is closely correlated with the upregulation of IAA biosynthesis and signal transduction.

### Iron homeostasis mediates physiological responses of *Arabidopsis* to SMF

**Figure 5:**
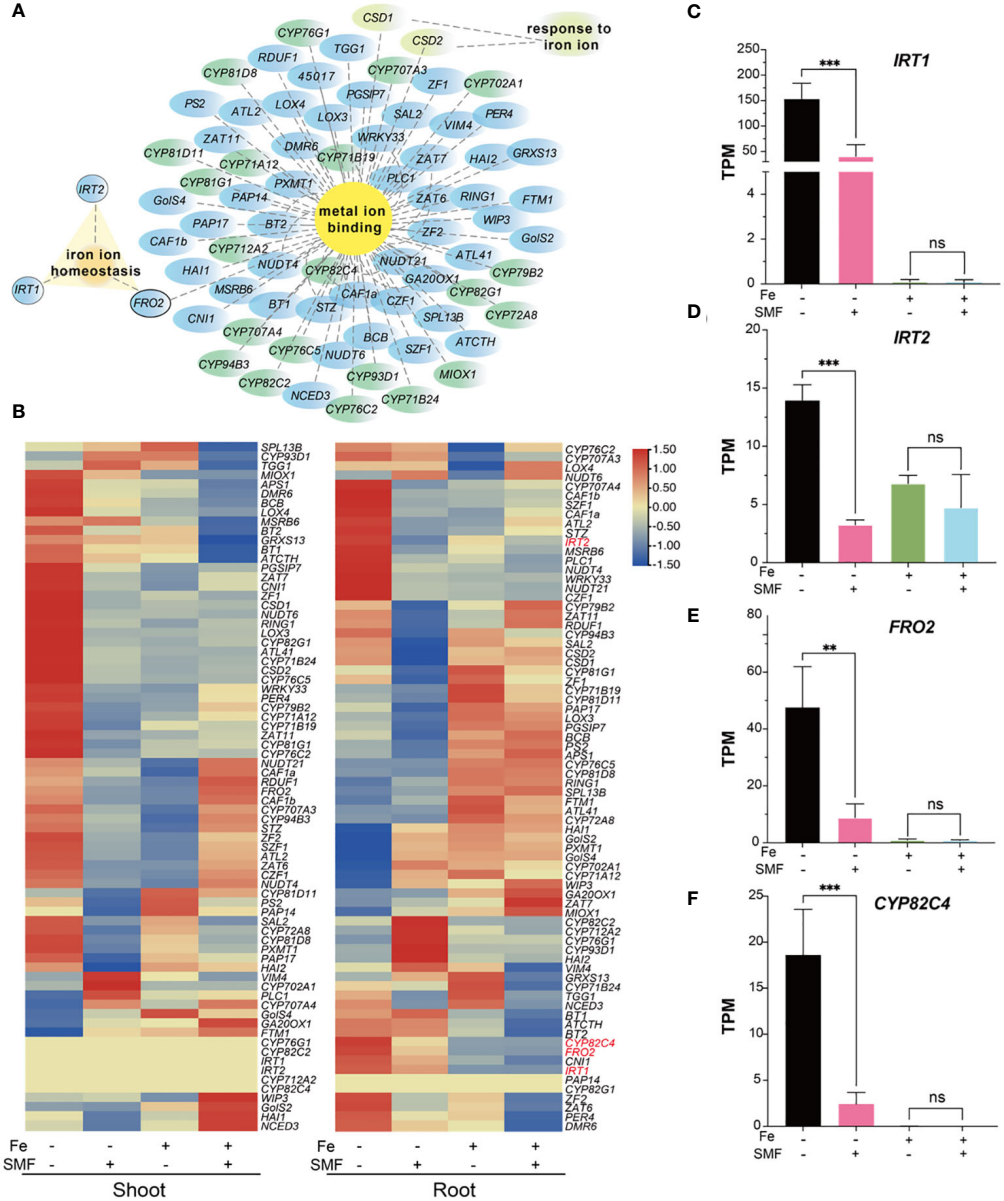
Transcriptional changes in DEGs involved in iron metabolism. **(A)** Network analysis of metal ion binding, respond to iron ion and iron ion homeostasis-related genes. **(B)** Heatmaps of normalized expression level represented by transcripts per million reads (TPM) of DEGs mentioned in **(A)**. **(C-F)** The comparison of TPM value of *IRT1* **(C)**, *IRT2* **(D)**, *FRO2* **(E)** and *CYP82C4* **(F)** in different conditions. The data were represented as mean ± SD of three biological replicates, whereas the asterisk indicated significant difference tested by one-way ANOVA, ns, no significance; **, *p*<0.01; ***, *p*<0.001.

Iron (Fe) is an essential micronutrient for plant growth and development as it involved in many fundamental biological processes including chlorophyll biosynthesis and energy transfer (Tripathi et al., 2018; Mahawar et al., 2023). As shown in **Figure 3**, many DEGs were enriched in biological processes such as metal ion binding, especially iron ion binding, suggesting that iron metabolism related genes played important roles in the physiological responses of plant to SMF. Cytoscape network analysis (**Supplementary Table S3**) revealed the expression pattern of all genes associated with metal ion binding, response to iron ion, and iron ion homeostasis in both shoot and root (**Figure 5**). For example, many genes of the P450 family have been identified in this study (**Figure 5A**, labeled green), which is consistent with previous study showing that P450 family is mainly involved in the iron ion binding and *CYP82C4* has been confirmed as an iron starvation-induced gene regulated by FIT in *Arabidopsis* (Murgia et al., 2011). Genes associated with iron homeostasis include *IRT1*, *IRT2*, *FRO2* were also identified to mediated MFE in *Arabidopsis* as well (**Figure 5A**).

A heatmap analysis was performed to better illustrate the expression pattern changes of iron metabolism related genes under different conditions. As shown in **Figure 5B**, 60% of iron metabolism genes were significantly downregulated in the shoot after SMF treatment. Meanwhile, iron stress also caused downregulation of many iron metabolism related genes. However, in the combination of SMF treatment and iron stress conditions, it is interesting that many iron stress mediated downregulated gene expressions were rescued by SMF in shoot. In root, the expression pattern was different under the same condition, suggesting that plants might adopt different regulatory mechanisms for iron homeostasis.

Three genes (*IRT1*, *IRT2, FRO2*) play essential roles in uptake of iron from soil and iron homeostasis, and *CYP82C4* expression in roots was strongly induced under iron deficiency (Rajniak et al., 2018). All these genes were found to be drastically downregulated in root by SMF treatment, also by iron stress, and by the combination of SMF exposure and iron stress as well in our study (**Figures 5B-F**).

Next, to verify whether the downregulation of these iron metabolism related genes could lead to iron content change in *Arabidopsis*, we measured the metal and iron content by ICP-OES and Ferrozine assay in shoots and roots, respectively (**Supplementary Figure S4**). Notably, iron stress led to a 7-fold increase in iron content in the roots and a 2-fold increase in the shoots, while Zn content decreased by 2 times in the roots after iron stress. The increased iron content in this condition is certainly possible because excessive iron was supplemented. However, in total, our data showed that no evident variation in metal contents including Fe, Cu, and Zn in *Arabidopsis* seedlings exposed to SMF compared with seedlings grown under sham conditions.

To further validate whether SMF plays roles in the regulation of iron homeostasis, *Arabidopsis irt1*, *irt2* and *fro2* mutants were grown under either SMF or sham conditions. All mutants showed inhibited growth under sham condition, probably due to the impaired iron uptake in these mutants (**Supplementary Figure S3**). It is worth pointing out that SMF exposure rescued the phenotype of these mutants, and the mutants showed similar growth including root length compared with that of the wild-type *Arabidopsis* seedlings. These results suggested that SMF treatment might help to maintain iron homeostasis thus reducing Fe requirements in *Arabidopsis.*

In addition, the Fe (III) and Cu (II) reductase activity of roots in plants grown under SMF and iron stress was also determined (**Supplementary Figure S4F, G**). The data showed that the activity of both enzymes was significantly increased by iron stress, but SMF treatment only had minor effects on the reductase activity.

### Effects of SMF on the antioxidant system in *Arabidopsis*

**Figure 6:**
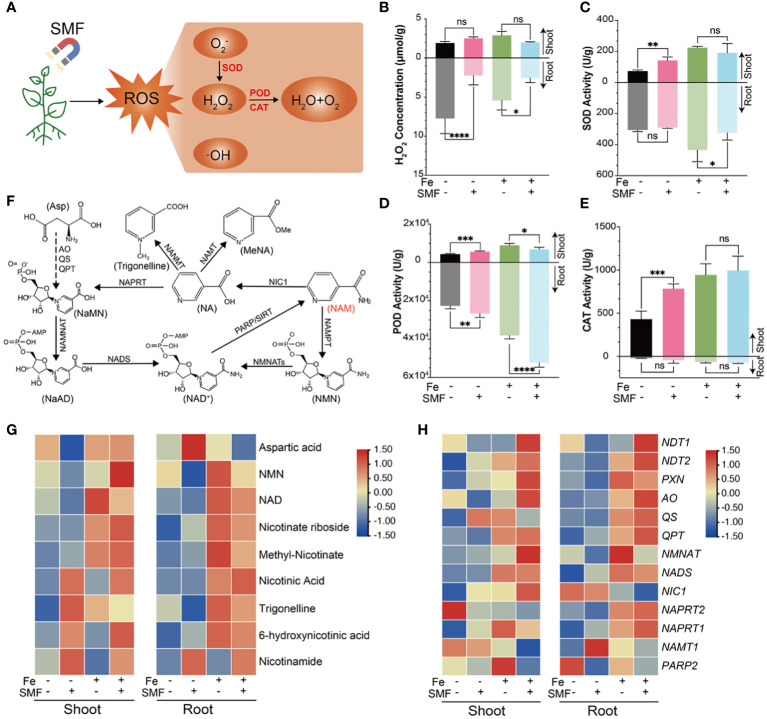
Oxidative stress and antioxidant changes of *Arabidopsis* seedlings upon SMF treatment, or iron stress, or the combination of SMF treatment and iron stress. **(A)** ROS-related metabolic pathways involved in *Arabidopsis* responding to SMF treatment. **(B-E)** The comparison of H_2_O_2_ concentration **(B)**, SOD **(C)**, POD **(D)**, CAT **(E)** activity in shoot and root of *Arabidopsis* seedlings upon SMF treatment, or iron stress, or the combination of SMF treatment and iron stress. The data were represented as mean ± SD of three biological replicates. whereas the asterisk indicated significant difference tested by one-way ANOVA, ns, no significance; *, *p*<0.05; **, *p*<0.01; ***, *p*<0.001; ****, *p*<0.0001. **(F)** Schematic diagram showing the NAD metabolism and Nicotinate conjugation in *Arabidopsis* (Wu et al., 2018). **(G)**The comparison of Differential Accumulation of Metabolites (DAMs) in NAD and NAM metabolism upon SMF treatment, or iron stress, or the combination of SMF treatment and iron stress. Relative abundance is expressed as log2 fold change. NAM, Nicotinamide; NAD, Nicotinamide adenine dinucleotide. **(H)** Heatmap of normalized expression level represented by TPM of DEGs involved in NAD and NAM metabolism pathway.

In plants, environmental and biological stresses generally lead to the accumulation of ROS and oxidative damage, which definitely affect many basic physiological processes such as photosynthesis and respiration. In response, plants utilize both non-enzymatic and enzymatic processes that synergistically maintain the balance of oxidants. The antioxidant defense system contributes to alleviating H_2_O_2_ and lipid peroxidative damage by increasing the production and activities of several antioxidant enzymes including APX, SOD, GR, CAT and POD. POD was found primarily in cellular peroxisomes and catalyzes the oxidation of diverse substrates with hydrogen peroxide as an electron acceptor. Both CAT and POD use iron (Fe) porphyrin as a prosthetic group to scavenge H_2_O_2_ to avoid oxidative damage. SOD is a metalloenzyme widely present in many species, which can convert superoxide anions and oxygen radicals into H_2_O_2_ and O_2_ (**Figure 6A**). APX, a heme peroxidase, detoxifies H_2_O_2_ (Liu et al., 2023).

Several studies reported that SMF exposure resulted in ROS level increased in plants (Shine et al., 2012; Haghighat et al., 2014). To investigate how *Arabidopsis* seedlings respond to SMF-induced oxidative stress and its underlying mechanism, H_2_O_2_ concentration of shoot and root was assessed, separately. Surprisingly, SMF treatment greatly decreased the content of H_2_O_2_ in the root, no matter in the presence or absence of iron stress. However, no significant change of H_2_O_2_ content was observed in the shoot upon SMF exposure (**Figure 6B**). This data strongly suggests that SMF induced antioxidant system involved in this process and was tissue specific.

Activities of several antioxidant enzymes (POD, SOD, CAT, and APX) were then assessed, and increased activities in shoots and/or roots under SMF exposure were observed (**Figures 6C-E**, **Supplementary Figure S5**). Moreover, SOD, POD and CAT activities were significantly enhanced by iron stress, and downregulated in the combination of SMF exposure and iron stress.

In addition to antioxidant enzymes, plants have many other metabolites involved in antioxidation as well. For example, nicotinamide (NAM) and nicotinic acid (NA) can protect plant cells from oxidative stress (Berglund et al., 2017). Therefore, metabolome analysis was performed by analyzing NAM and NA-related metabolites and related genes (**Figures 6F-H**). Our data revealed specific accumulations of NA, NAM, Trigonelline and 6-hydroxy-NA in the shoot, with only NAM accumulating in the root after SMF exposure (**Figure 6G**). In contrast, the accumulation of most related metabolites in both shoot and root was significantly increased under iron stress and further enhanced in the combination of SMF exposure and iron stress conditions (**Figure 6G**).

Studies have shown that NAM, NA and Trigonelline play an important role as antioxidants (Ponrasu et al., 2013; Antonisamy et al., 2016; Rehan S et al., 2022). We also analyzed the gene expression profile related to the NAM pathway. Although no significant differences were observed under SMF exposure, similarly, their expression was upregulated in both shoot and root under iron stress and further increased in the combination of SMF exposure and iron stress conditions (**Figure 6H**).

Taken together, iron stress, as well as other environmental stresses as reported previously, seems to result in tissue specific ROS level changes in plants. Plant has different strategies to be more resistant to the oxidative stress. A specific magnetic field exposure as demonstrated in this study, leads to enhanced antioxidant enzyme activities, upregulated NAM pathway related genes expression, and accumulation of NAM and NA-related metabolites, which certainly facilitates the plant to reduce and maintain appropriate ROS level.

## Discussion

While the MFEs on plant growth and development have been extensively documented and discussed (Samani et al., 2013; Maffei, 2014; Vashisth and Joshi, 2017; Bahadir et al., 2018; Jin et al., 2019; Sarraf et al., 2020; Podleśny et al., 2021; Naseer et al., 2022; Abhary and Akhkha, 2023; Hafeez et al., 2023), the underlying molecular mechanism remains largely unclear. Here in this study, we evaluated different magnetic setups for their effects on *Arabidopsis* and identified that triangular prism magnets perpendicular to the direction of gravity have most significant effects on growth, regardless of whether it is the North or South pole. The biological effects of high-gradient magnetic fields have gained the increased attention of scientists in recent years, however, how a gradient magnetic field could affect biological systems remains unknown (Zablotskii et al., 2016). There could be couple of possible mechanisms. Firstly, gradient magnetic field may have better chance to change the probability of ion-channel on/off switch than uniform magnetic field. Secondly, the magnetic force acting on a magnetic dipole moment is proportional to the field gradient, therefore, if the MFE is mediated by biomolecules with innate magnetic moment (e.g. MagR), gradient magnetic field could have more significant effects compared with uniform magnetic fields. However, it is still an open question at this stage.

SMF exposure can increase the biomass, relative leaf area, root length and lateral root number of *Arabidopsis*, however, the chlorophyll content was not affected, which is inconsistent with the findings reported by Abdollahi (Abdollahi et al., 2019). Meanwhile, iron stress could inhibit plant growth and increase chlorophyll contents in our study. It is interesting to point out that SMF treatment could partially rescue the iron stress induced growth inhibition in *Arabidopsis* seedlings.

Thus, to investigate the mechanism of MFE on plants and elucidate the possible connection between MFE and iron metabolism, a comparative analysis of both the gene expression profiles and metabolism was then performed. Most DEGs were enriched in biological processes such as auxin-related pathways, including biosynthesis and signal transduction, antioxidant metabolism and metal ion metabolism also enriched. Auxin are plant hormones playing essential roles in plant growth and development across different environmental conditions. Several major classes of auxin-related genes are identified in plants. In our study, the expression of auxin synthesis genes (*TAA1*, *YUC2/4*) in the shoot and auxin transporter genes (*PIN1/3/6*) in the root were upregulated by SMF treatment, and were further enhanced in the combination of SMF exposure and iron stress. The expression of many genes from the Aux/IAA family was also induced by SMF treatment. For example, *IAA10*, *IAA5*, *IAA6*, *IAA20*, *IAA19*, *IAA3*0 and *IAA31* were induced by SMF in the shoot, and *IAA19*, *IAA29*, *IAA30* in the root. In addition, *AtGH3.9* which has been proposed to maintain auxin homeostasis in stress adaptation responses in *Arabidopsis* (Park et al., 2007), was significantly induced after SMF treatment, indicating that auxin homeostasis may play a role to response the environmental stress from external magnetic field in plants.

Iron (Fe) is an essential micronutrient for plants. It is a cofactor of more than 300 enzymes and plays an irreplaceable role in many fundamental biological processes such as respiration and photosynthesis (Kim et al., 2019). However, excessive iron can be toxic and lead to the production of high levels of ROS, which can damage cells and even cause cell death. Thus, the iron metabolism was tightly regulated in plants including *Arabidopsis* to maintain iron homeostasis, thus to avoid both iron deficiency and iron toxicity (Connorton et al., 2017). Iron uptake is essential in iron homeostasis since iron is mainly obtained through direct uptake from the soil by roots, and several key genes including *FRO2*, *IRT1* and *IRT2* are involved in iron uptake and iron transport in *Arabidopsis* (Kobayashi and Nishizawa, 2012). *AtCYP82C4*, an iron deficiency inducer, was significantly down-regulated after SMF treatment in roots, indicating that SMF treatment reduced the iron requirement of *Arabidopsis* seedlings. *AtMIOX1*, encoding Myo-inositol oxygenase (MIOX) which catalyzes the cleavage of myo-inositol to UDP-glucuronic acid and mediates plant’s adaptation to abiotic stress factors such as alkaline stress (Chen et al., 2015; Guo et al., 2023), showed different expression profiles in the shoots and roots. The expression of *AtMIOX1* was down-regulated in the shoots and up-regulated in the roots after SMF treatment, and the molecular mechanism needs to be further explored.

Our results showed that SMF treatment may have different effects in shoots and roots of *Arabidopsis*. In shoot, both SMF exposure and iron stress caused downregulation of many iron metabolism related genes, but in the combination of SMF treatment and iron stress conditions, these downregulations were partially reversed and rescued. In contrast, the expression pattern of iron metabolism related genes was different and more complicated in the same condition in root. It is important to point out that the expression of all three genes involved in iron uptake in root are drastically inhibited by SMF treatment, or by iron stress conditions, or by the combination of SMF treatment and iron stress. However, there is almost no change in iron content in plant tissues, suggesting plants may have other mechanisms to maintain iron homeostasis.

*Arabidopsis irt1*, *irt2* and *fro2* mutants showed inhibited growth, probably due to impaired iron uptake. However, SMF exposure rescues the inhibited growth in these mutants, which unambiguously demonstrated that SMF exposure facilitates plants to maintain iron homeostasis thus reduced Fe requirements.

In plants, various environmental and biological stresses result in ROS accumulation and oxidative damage, and plants have developed different strategies including both non-enzymatic and enzymatic processes to synergistically maintain oxidant balance (Pinto et al., 2019; Liu et al., 2023). The effect of magnetic fields on ROS level seems very complicated and is still controversial based on previous studies on animals and plants (Wang and Zhang, 2017). Both increased and decreased ROS levels upon magnetic field exposure have been reported. For example, exposure to increased magnetic fields caused the accumulation of ROS and the alteration of oxidative enzyme activities (Maffei, 2014), and inhomogeneous SMF exposure decreased ROS level (Csillag et al., 2014). In our study, SMF treatment greatly reduced the ROS level in the root, this finding is inconsistent with the results of previous studies (Cakmak et al., 2012; Jouni et al., 2012; Zareei et al., 2019).

There could be many reasons to explain the discrepancies. First of all, the differences in magnetic field type and strength could have different effects on ROS level. In our study, we used a regular triangular prism magnet perpendicular to the direction of gravity to produce a highly inhomogeneous SMF, which is unique in magnetic field treatment. Secondly, we determined the ROS level in shoot and root separately, which would give us more precise results of the tissue specific ROS level. Furthermore, increased activity of several antioxidant enzymes (POD, SOD, CAT, and APX) upon SMF exposure was observed, as well as specific accumulations of NA, NAM, Trigonelline and 6-hydroxy-NA in the shoot and NAM accumulation in the root under SMF treatment. These metabolites function as important antioxidants to regulate various physiologic processes (Ponrasu et al., 2013; Antonisamy et al., 2016; Rehan S et al., 2022). Our data collectively suggested SMF exposure facilitated the plants to reduce ROS levels and synergistically maintain the oxidant balance. It is possible that SMF exposure may enhance electron transport in *Arabidopsis* seedlings, thereby activating the ROS signaling pathway (Zhao et al., 2020) and promoting the accumulation of antioxidants.

In conclusion, this study has revealed the effects and possible regulatory mechanism of SMF on *Arabidopsis*. Using a unique inhomogeneous SMF generated by a regular triangular prism magnet perpendicular to the direction of gravity, we showed that SMF sinificantly promote the plant growth, as shown by increased the biomass, relative leaf area, root length and lateral root number in *Arabidopsis*. Our work uncovered the regulation of SMF on IAA biosynthesis and signal transduction in A*rabidopsis*, which is consistent with the promoted growth upon SMF exposure. We also demonstrated that SMF treatment facilitated plants to maintain the iron homeostasis thus reducing Fe requirements, and SMF exposure also helped the plants to reduce ROS level and synergistically maintain the oxidant balance, which could be beneficial for plant survival and growth. Understanding the underlying mechanism of magnetic field effects on plants and the related regulatory network would provide insight into the development of novel plant synthetic biology technologies in order to engineer stress-resistant and high-yielding crops.

## Data availability statement

The datasets presented in this study can be found in online repositories. The names of the repository/repositories and accession number(s) can be found below: https://www.ncbi.nlm.nih.gov/, PRJNA1071296.

## Author contributions

XZ: Conceptualization, Data curation, Formal analysis, Investigation, Methodology, Project administration, Resources, Software, Validation, Visualization, Writing – original draft, Writing – review & editing. LZ: Methodology, Resources, Writing – review & editing. PZ: Formal analysis, Methodology, Writing – review & editing. HX: Formal analysis, Methodology, Resources, Writing – review & editing. JS: Formal analysis, Investigation, Methodology, Writing – review & editing. YC: Formal analysis, Methodology, Writing – review & editing. TC: Conceptualization, Formal analysis, Funding acquisition, Project administration, Resources, Supervision, Visualization, Writing – original draft, Writing – review & editing. CX: Conceptualization, Funding acquisition, Project administration, Writing – review & editing.

## Supplementary material

Supplementary Figure 1 Schematic diagram and magnetic field lines simulation of a triangular prism magnet.

Supplementary Figure 2 Chlorophyll contents of *Arabidopsis* in different conditions.

Supplementary Figure 3 The growth of *Arabidopsis* iron uptake mutants upon SMF treatment.

Supplementary Figure 4 Measurements of metal ion content in shoot and root of *Arabidopsis* seedlings, as well as the activity of Fe (III) and Cu (II) reductase in roots upon SMF treatment, iron stress or the combination of SMF treatment and iron stress.

Supplementary Figure 5 APX activity in shoot and root of *Arabidopsis* seedlings upon SMF treatment, iron stress or the combination of SMF treatment and iron stress.
