# Supplementary material for: Comparative transcriptomic analysis revealed important processes underlying the static magnetic field effects on Arabidopsis
Source: Front Plant Sci. 2024 May 28;15:1390031. doi: 10.3389/fpls.2024.1390031 (PMC11165219; doi:10.3389/fpls.2024.1390031)
Supplement: Supplementary Figure 1 — Schematic diagram and magnetic field lines simulation of a triangular prism magnet. [file DataSheet_1.pdf]

Supplementary Material

Comparative transcriptomic analysis revealed important processes underlying the static magnetic field effects on *Arabidopsis*

Xiujuan Zhou, Lin Zhang, Peng Zhang, Hang Xu, Jialei Song, Yafei Chang, Tiantian Cai*, Can Xie*

*** Correspondence:** Tiantian Cai: tiantiancai@zju.edu.cn; Can Xie: canxie@zju.edu.cn

1 Supplementary Figures and Tables

1.1 Supplementary Figures

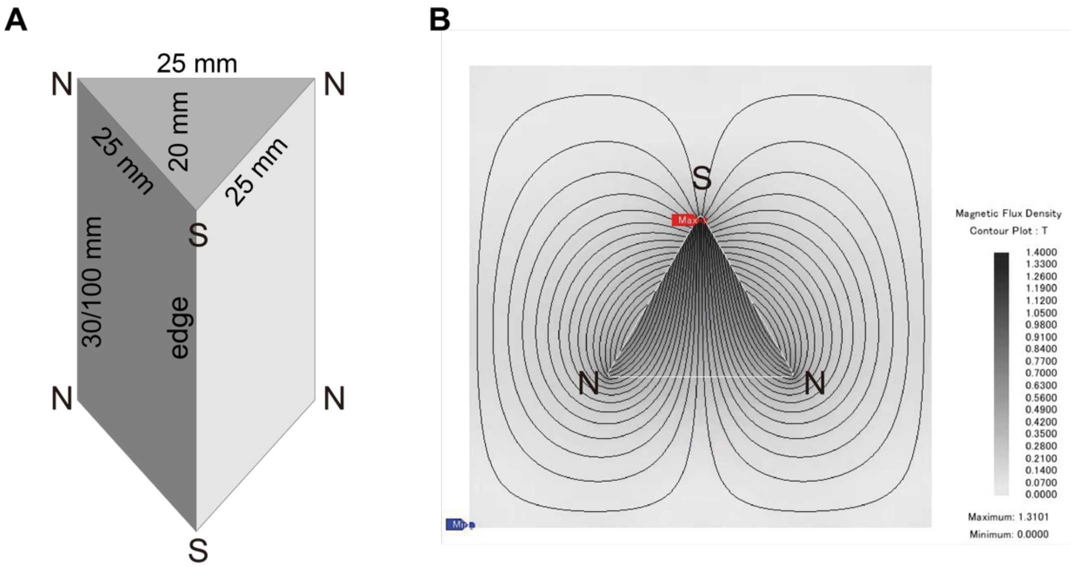

Supplementary Figure 1. Schematic diagram and magnetic field lines simulation of a triangular prism magnet. (A) Schematic diagram of a 3D model of a triangular prism magnet. When the edge is the S pole, the other two ends are the N pole (as shown, labeled as S-type in Figure 1O, P, S and T). Similarly, if the edge is N pole, the other two ends are the S pole (not shown, labeled as N-type in Figure 1O, P, S and T). **(B)** Magnetic field lines simulation of a triangular prism magnet.

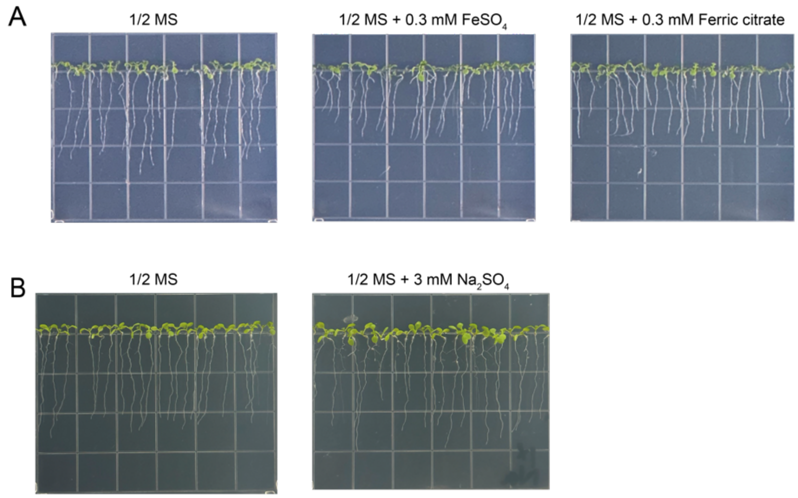

Supplementary Figure 2. The growth inhibition of *Arabidopsis* seedlings upon $FeSO_4$ treatment is due to iron stress, but not from sulfate. (A) Representative images of the seedlings after 7 days of 0.3 mM $FeSO_4 \cdot 7H_2O$ and Ferric citrate treatment. (B) Representative images of the seedlings after 7 days of 3 mM Na_2SO_4 treatment.

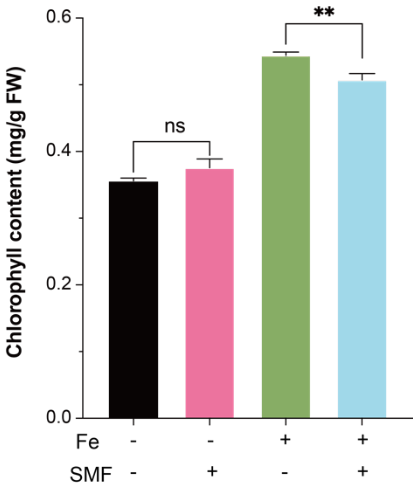

Supplementary Figure 3. Chlorophyll contents of *Arabidopsis* in different conditions. FW, fresh weight. The data were represented as mean ± SD of three biological replicates, whereas the asterisk indicated significant difference tested by one-way ANOVA, ns, no significance; **, $p<0.01$.

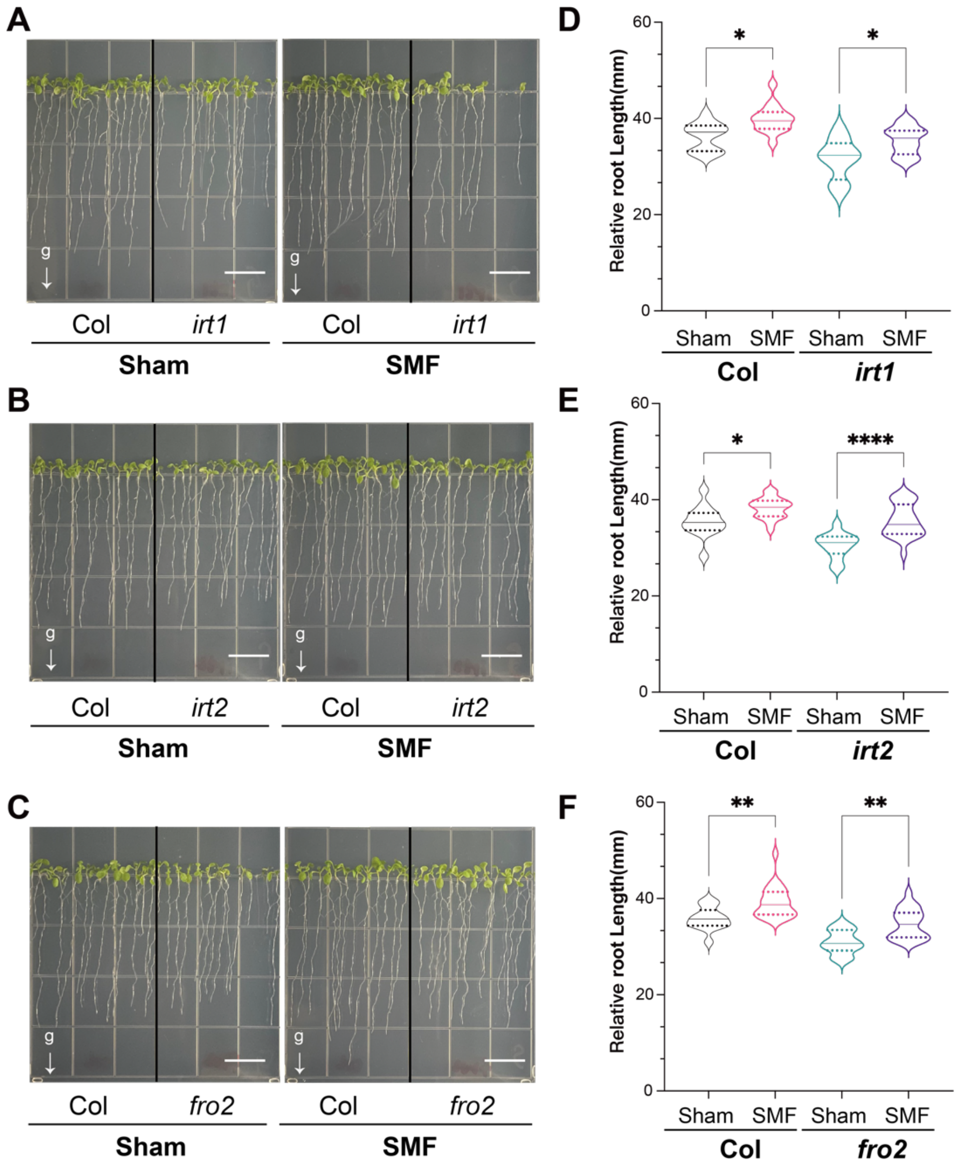

Supplementary Figure 4. The growth of *Arabidopsis* iron uptake mutants upon SMF treatment. (A-C) Representative images of the mutants *irt1* **(A),** *irt2* **(B)** and *fro2* **(C)** seedlings after 7 days of SMF treatment. **(D-F)** The relative root length of the mutants *irt1* **(D),** *irt2* **(E)** and *fro2* **(F)** seedlings after 7 days of SMF treatment. significant difference tested by one-way ANOVA, ns, no significance; *, $p<0.05$; **, $p<0.01$; ****, $p<0.0001$.

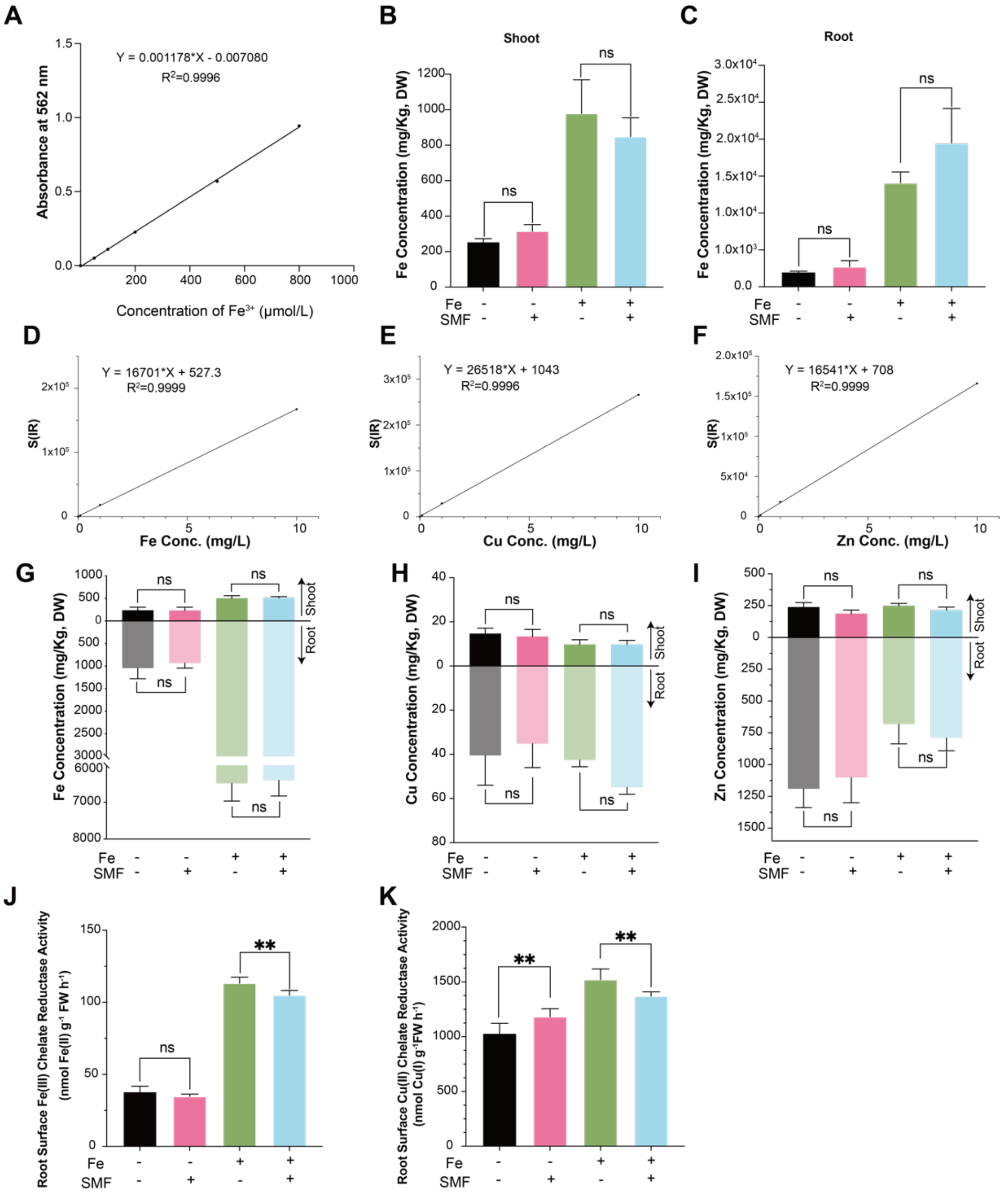

Supplementary Figure 5. Measurements of metal ion content in shoot and root of *Arabidopsis* seedlings, as well as the activity of Fe (III) and Cu (II) reductase in roots upon SMF treatment, iron stress or the combination of SMF treatment and iron stress. (A) Standard curve for ferrozine assay. **(B, C)** Iron concentrations in the shoot **(B)** and root **(C)** of *Arabidopsis* seedlings were measured after grown under sham or SMF with or without iron stress using ferrozine assay. **(D-F)** Standard curve of Fe (D), Cu (E), Zn (F) for ICP-OES. **(G-I)** The concentrations of Fe **(G)**, Cu **(H)**, and Zn **(I)** (expressed as mg/Kg DW) in the shoot and root of *Arabidopsis* seedlings grown under sham or SMF with or without iron stress conditions determined by ICP-OEs. DW: dry weight. **(J, K)** In vivo root ferric reductase activity **(J)** and cupric reductase activity **(K)** of *Arabidopsis* grown under sham or SMF with or without iron stress conditions. The data were represented as mean ± SD of three biological replicates, whereas the asterisk indicated significant difference tested by one-way ANOVA, ns, no significance; **, $p<0.01$.

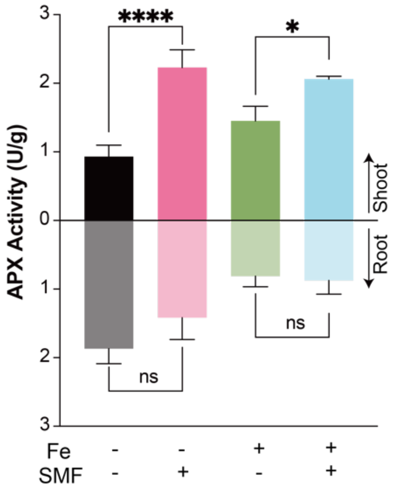

Supplementary Figure 6. APX activity in shoot and root of *Arabidopsis* seedlings upon SMF treatment, iron stress or the combination of SMF treatment and iron stress. The data were represented as mean ± SD of three biological replicates, whereas the asterisk indicated significant difference tested by one-way ANOVA, ns, no significance; *, p<0.05; ****, p<0.0001.

1.2 Supplementary Tables

Supplementary Table 1. Summary of read statistics from RNA-sequencing.

	Samples	Raw reads	Clean reads	Error rate (%)	Q20(%)	Q30(%)
Shoot	Sham1	43721458	43443130	0.0235	98.66	95.76
	Sham2	44651838	44351184	0.024	98.47	95.24
	Sham3	48907124	48570146	0.0237	98.56	95.51
	SMF1	52903134	52519174	0.0236	98.6	95.64
	SMF2	44981750	44621734	0.0236	98.62	95.69
	SMF3	50838366	50524842	0.0238	98.52	95.37
	Sham with Fe1	45334662	44981116	0.0236	98.62	95.65
	Sham with Fe2	43191642	42900706	0.0237	98.56	95.49
	Sham with Fe3	50457526	50086966	0.0236	98.61	95.65
	SMF with Fe1	49178722	48835896	0.0237	98.58	95.56
	SMF with Fe2	47023902	46741688	0.0237	98.55	95.47
	SMF with Fe3	45217820	44892794	0.0239	98.49	95.32
Root	Sham1	43546714	43251112	0.0237	98.54	95.5
	Sham2	47702158	47380512	0.0239	98.47	95.3
	Sham3	59678674	59251230	0.0239	98.48	95.33
	SMF1	47195082	46848948	0.0238	98.54	95.5
	SMF2	43961844	43612298	0.0239	98.49	95.36
	SMF3	50974776	50433526	0.0242	98.35	95.16
	Sham with Fe1	41700036	41406406	0.0238	98.52	95.41
	Sham with Fe2	43122040	42783150	0.0236	98.6	95.69
	Sham with Fe3	48849982	48495302	0.024	98.45	95.24
	SMF with Fe1	57510750	57153304	0.0238	98.54	95.44
	SMF with Fe2	47077272	46766260	0.0237	98.55	95.51
	SMF with Fe3	50972550	50635650	0.0236	98.61	95.68

Supplementary Table 2. Summary statistics of UniGene functional annotations.

Database	Number	Percentage (%)
GO	25027	92.78
KEGG	10099	37.44
COG	24407	90.48
NR	25961	96.24
Swiss-Prot	20948	77.65
Pfam	20856	77.31
Total_anno	26040	96.53

Supplementary Table 3. List of the DEGs in metal ion metabolism for gene network analysis.

Type	Gene	Pvalue
metal ion binding	CYP79B2	0.01812474
metal ion binding	CYP707A3	0.01812474
metal ion binding	CYP81D11	0.01812474
metal ion binding	CYP81D8	0.01812474
metal ion binding	CYP71B24	0.01812474
metal ion binding	CYP76C5	0.01812474
metal ion binding	CYP707A4	0.01812474
metal ion binding	CYP72A8	0.01812474
metal ion binding	CYP81G1	0.01812474
metal ion binding	CYP76C2	0.01812474
metal ion binding	CYP82G1	0.01812474
metal ion binding	CYP71B19	0.01812474
metal ion binding	MIOX1	6.14E-04
metal ion binding	CYP712A2	6.14E-04
metal ion binding	CYP82C4	6.14E-04
metal ion binding	CYP76G1	6.14E-04
metal ion binding	CYP94B3	6.14E-04
metal ion binding	CYP702A1	6.14E-04
metal ion binding	CYP82C2	6.14E-04
metal ion binding	CYP71A12	6.14E-04
metal ion binding	CYP93D1	6.14E-04
iron ion homeostasis	IRT1	0.036369296
iron ion homeostasis	FRO2	0.036369296
iron ion homeostasis	IRT2	0.036369296
metal ion binding	ZAT7	0.012911929
metal ion binding	ZAT7	0.012911929
metal ion binding	ZF1	0.012911929
metal ion binding	GA20OX1	0.012911929
metal ion binding	SZF1	0.012911929
metal ion binding	CSD2	0.012911929
metal ion binding	SAL2	0.012911929
metal ion binding	LOX3	0.012911929
metal ion binding	WRKY33	0.012911929
metal ion binding	NUDT6	0.012911929
metal ion binding	PLC1	0.012911929
metal ion binding	BT2	0.012911929
metal ion binding	45017	0.012911929
metal ion binding	BCB	0.012911929
metal ion binding	FTM1	0.012911929

metal ion binding	PER4	0.012911929
metal ion binding	DMR6	0.012911929
metal ion binding	PGSIP7	0.012911929
metal ion binding	CNI1	0.012911929
metal ion binding	ZF2	0.012911929
metal ion binding	VIM4	0.012911929
metal ion binding	ZAT11	0.012911929
metal ion binding	ATL41	0.012911929
metal ion binding	PS2	0.012911929
metal ion binding	ZAT6	0.012911929
metal ion binding	PAP17	0.012911929
metal ion binding	LOX4	0.012911929
metal ion binding	RING1	0.012911929
metal ion binding	CZF1	0.012911929
metal ion binding	PAP14	0.012911929
metal ion binding	CSD1	0.012911929
metal ion binding	MSRB6	0.007509154
metal ion binding	GolS4	0.007509154
metal ion binding	WIP3	0.007509154
metal ion binding	NUDT21	0.007509154
metal ion binding	CAF1a	0.007509154
metal ion binding	HAI2	0.007509154
metal ion binding	HAI1	0.007509154
metal ion binding	PXMT1	0.007509154
metal ion binding	NUDT4	0.007509154
metal ion binding	GolS2	0.007509154
metal ion binding	STZ	0.007509154
metal ion binding	FRO2	0.007509154
metal ion binding	GA20OX1	0.02775373
metal ion binding	DMR6	0.02775373
metal ion binding	CAF1b	0.02775373
metal ion binding	ZF2	0.02775373
metal ion binding	NUDT4	0.02775373
metal ion binding	WRKY33	0.02775373
metal ion binding	BT1	0.02775373
metal ion binding	STZ	0.02775373
metal ion binding	ZAT6	0.02775373
metal ion binding	RDUF1	0.02775373
metal ion binding	NCED3	0.02775373
metal ion binding	NUDT21	0.02775373
metal ion binding	CAF1a	0.02775373
metal ion binding	SPL13B	0.02775373

metal ion binding	CZF1	0.02775373
metal ion binding	ATL2	0.02775373
metal ion binding	BT2	0.02775373
metal ion binding	GRXS13	0.02775373
metal ion binding	ATCTH	0.037859261
metal ion binding	TGG1	0.037859261
response to iron ion	CSD2	0.009855671
response to iron ion	CSD1	0.009855671
